# Action of Iodide of Potassium upon the Elimination of Lead

**Published:** 1880-11

**Authors:** 


ACTION OF IODIDE OF POTASSIUM UPON THE ELIMINATION OF LEAD.

At the request of Prof. Vulpin, Dr. Gabriel Pouchet, of the chemical laboratory of the Faculty of Paris, examined by quantitative analysis of the urine of patients suffering from saturnism, the effect of the administration of iodide of potassium on the elimination of lead. The experiments were of an electrolytic nature of which the details would not interest the practitioner. Suffice it to say, the specimens of urine examined always measured from five to ten pints, quantities sufficiently large to permit the detection of even the smallest traces of the noxious metal. Among all the afflicted during the time of aggravated symptoms the urine contained an average of one milligramme of metallic lead to the litre of urine examined.

Under the influence of iodide of potassium given in doses of four to six grammes daily, the elimination of lead increases quickly, but again decreases at the end of from six to ten days when it becomes less marked than before the treatment was instituted. During the first days of treatment a rapid desaturation of the system which is more or less decided according to the intensity of the morbid phenomena. Thus in the case of a patient who was gravely afflicted, the quantity of lead passed by the urine, immediately after the administration of the iodide, rose to five milligrammes per litre.

After the continuous exhibition of the salt for more than two weeks, the elimination of lead ceases almost altogether, so that in fifteen litres of urine only a trace of lead was discernible. But if after withdrawing the remedy, the patient is allowed to rest some days, prior to the readministration of the iodide, small quantities of lead will again be eliminated with the urine. Hence arises the therapeutic indication of employing the iodides for a long time and instituting, however, intervals of repose during which the remedy is not to be exhibited.

M. Pouchet also analyzed the urine of a patient treated exclusively with bromide of potassium without detecting any increase of the lead eliminated. From this he deducts the inefficiency of the bromide in the treatment of leading poisoning.—*Arch. de phys. norm. et pathol.—Cincinnati Lancet and Clinic.*

